# Cubic Fe-bearing majorite synthesized at 18–25 GPa and 1000 °C: implications for element transport, subducted slab rheology and diamond formation

**DOI:** 10.1038/s41598-023-43037-6

**Published:** 2023-09-22

**Authors:** Vincenzo Stagno, Luca Bindi, Barbara Bonechi, Steeve Greaux, Sonja Aulbach, Tetsuo Irifune, Stefano Lupi, Giulia Marras, Catherine A. McCammon, Manuela Nazzari, Federica Piccirilli, Brent Poe, Claudia Romano, Piergiorgio Scarlato

**Affiliations:** 1 Dipartimento di Scienze Della Terra, Sapienza Università di Roma, Rome, Italy; 2 Dipartimento di Scienze Della Terra, Università di Firenze, Firenze, Italy; 3 University of Manchester, Manchester, UK; 4 Geodynamic Research Center, Ehime University, Matsuyama, Japan; 5 Earth-Life Science Institute, Tokyo Institute of Technology, Tokyo, Japan; 6 Institute for Geosciences, Frankfurt Isotope & Element Research Center (FIERCE), Goethe University, Altenhöferallee 1, 60438 Frankfurt, Germany; 7 Department of Physics, Sapienza University of Rome, Rome, Italy; 8 Bayerisches Geoinstitut, Bayreuth Universität, Bayreuth, Germany; 9 Istituto Nazionale di Geofisica e Vulcanologia, Rome, Italy; 10 CNR-IOM, Area Science Park, 34012 Trieste, Italy; 11 Dipartimento di Scienze, Università Di Chieti, Chieti, Italy; 12 Dipartimento di Scienze, Università Di Roma Tre, Rome, Italy

**Keywords:** Mineralogy, Petrology

## Abstract

The chemistry and mineralogy of slabs subducted into lower mantle control slab rheology and impact the deep volatile cycle. It is known that the metamorphism of little-altered oceanic crust results in eclogite rocks with subequal proportions of garnet and clinopyroxene. With increasing pressure, these minerals react to stabilize pyrope-rich tetragonal majoritic garnet. However, some eclogites contain higher proportions of omphacitic clinopyroxene, caused by Na- and Si-rich metasomatism on the ocean floor or during subduction. The mineralogy of such eclogites is expected to evolve differently. Here, we discuss the results of the crystallization products of omphacitic glass at ~ 18 and ~ 25 GPa and 1000 °C to simulate P–T regimes of cold subduction. The full characterization of the recovered samples indicates evidence of crystallization of Na-, Si-rich cubic instead of tetragonal majorite. This cubic majorite can incorporate large amounts of ferric iron, promoting redox reactions with surrounding volatile-bearing fluids and, ultimately, diamond formation. In addition, the occurrence of cubic majorite in the slab would affect the local density, favoring the continued buoyancy of the slab as previously proposed by seismic observations. Attention must be paid to omphacitic inclusions in sublithospheric diamonds as these might have experienced back-transformation from the HP isochemical cubic phase.

## Introduction

The fate of the oceanic lithosphere’s chemical budget during subduction is linked to the complex motion and morphology of the subducting slab, linked to trench migration rate and slab-dip angle that determine the conditions for slab penetration at depth^1^. Density and viscosity of the subducting slab are key variables that control positive buoyancy vs. sinking processes at depth^2–6^. Knowledge of these variables allows a better understanding of the origin of deep-focus earthquakes, generally defined as seismic events with hypocenters located at depths greater than 350 km^7^. To date, such events have been interpreted as due to shear instabilities associated with polymorphic transformations of olivine (Mg,Fe)_2_SiO_4_, the most abundant mineral in the upper mantle (UM), to wadsleyite/ringwoodite with increasing depth, accompanied by positive density changes of 6% and 2%, respectively^8^. In subduction zones, this transformation of olivine can be promoted at shallower depths under lower temperature regimes, and this may affect the dynamics of the subducting slab and the origin of deep-focus earthquakes^9–11^. According to the common mantle adiabat, where temperatures are expected to be ~ 1500 °C in the transition zone (TZ)^12^, olivine transforms to wadsleyite at a depth of 410 km, to ringwoodite at a depth of 520 km, and finally to (Mg,Fe)SiO_3_ bridgmanite + (Mg,Fe)O (magnesio-wüstite) at a depth of about 660 km^13^. Subducting oceanic crust is also exposed to important phase transitions and reactions, such as clinopyroxene (*Cpx*) plus garnet (*Grt*) contained in the eclogitic portions of the slab initially roughly at 1:1 volume ratio^14^ forming majoritic garnet (*Maj*). In turn, the decomposition of *Maj* under anhydrous conditions leads to the formation of both calcic (i.e., davemaoite) and ferromagnesian (i.e., bridgmanite) silicate perovskites plus additional minor phases like stishovite, NAL (new aluminous) and CF (calcium-ferrite) phases. The presence of such phases is either substantiated by experimental studies^15,16^ or corroborated directly by the analysis of mineral inclusions present in sub lithospheric diamonds^17,18^. Among the several mineral phase transformations occurring during subduction, the continuous reaction of eclogitic *Cpx* (i.e. omphacite) with *Grt* to form *Maj* is of particular interest because of its use as a geobarometer for sub lithospheric diamonds^19^, and the associated increase in slab density (~ 6%; ref.^20^) at about 350–450 km. The transformations *Cpx* + *Grt* → *Cpx* + *Maj* → *Maj* should occur over a broader depth interval, by about an order of magnitude, than those involving olivine^21–25^. The effect on buoyancy forces by the majorite reactions occurs over a large P-interval relative to the olivine-waddsleyite-ringwoodite transitions. During subduction, omphacitic *Cpx* dissolves progressively into *Grt* within a pressure range of 7–17 GPa, to form *Maj*^16,26^. The occurrence of majoritic *Grt* included in sub-lithospheric diamonds along with the frequent report of E-type inclusions in diamonds suggest the potential role of the involved Fe-bearing minerals (i.e., *Cpx*, *Grt* and *Maj*) to buffer the local redox conditions and promote diamond precipitation through redox reactions at the expense of oxidized CO_2_-bearing fluids^27–29^. Eclogite xenoliths exhumed from the mantle are commonly interpreted as having subducted oceanic crust precursors^30^. These rocks are characterized by chemical and mineralogical variability resulting from variations in the nature of the protolith (various portions of oceanic crust) as well as interaction with metasomatic fluids (on the ocean floor, during subduction and in the mantle)^31^. Experimental studies, observations in nature and thermodynamic modelling suggest that typical oceanic crust metamorphoses to eclogite with subequal modal abundances of *Grt* and omphacitic *Cpx*^30–32^. However, some eclogitic xenoliths have been reported to contain much more *Cpx* than *Grt*, up to almost 80%^33,34^, and jadeitites and omphacitites are minor lithologies that are often reported from high-pressure-low-temperature metamorphic terranes^35^. As a consequence, subduction of very *Cpx*-rich lithologies may result in incomplete dissolution in garnet to form majorite, resulting in retention of excess omphacitic cpx at very high pressures.

To date, experimental studies on the stability of omphacitic *Cpx* at high pressure and temperature are sparse in the literature and arrive at two main contrasting results: (1) decomposition to an assemblage of tetragonal Na-bearing *Maj* + Ca-perovskite + stishovite, or (2) formation of a post-*Cpx* phase^36,37^. In detail, Bobrov et al.^38–40^ proved that most of the Na present in majoritic *Grt* is accommodated via the pressure dependent exchange reaction Na^+^ + Si^4+^ = Mg^2+^ + Al^3+^ (Na for Mg in the *X* site and Si for Al in the *Y* site). Such a mechanism of sodium incorporation in tetragonal majoritic *Grt* supported the idea of the presence of a Na-*Maj* end member in *Grt* solid solution^41,42^. The Na_2_MgSi_5_O_12_ end-member, indeed, was later synthesized in the model system Mg_3_Al_2_Si_3_O_12_–Na_2_MgSi_5_O_12_ at 17.5 GPa and 1700°C^43^ and structurally characterized by Bindi et al.^44^. These authors confirmed the ability of majoritic *Grt* to incorporate significant concentrations of Na and Si as previously reported by authors^45,46^ who experimentally observed the immiscibility between Al-rich *Maj* and Na, Si-rich *Maj* (along with other minerals) between 13 and 15 GPa at 1550–1700 °C in the Na_2_O-CaO-FeO-MgO-Al_2_O_3_-SiO_2_ (NCFMAS) system. The finding of majorite with both peridotitic and eclogitic affinity as inclusion in diamonds have raised important considerations on the role that this mineral might have in diamond formation processes at expenses of C-saturated fluids^27–29^. Therefore, mineralogical and petrological evidence of the existence of Na, Si-rich *Maj* would imply an important role as host for Na and diamond formation from an alkali-silica rich growth medium in the lower part of the upper mantle (UM) and transition zone (TZ).

We here report the synthesis, using the multi anvil press technique, of Na-, Si-rich Fe-bearing *Maj* from a starting glass with Na-, Si-rich omphacitic composition (see Table S1) with a cubic structure rather than the common tetragonal symmetry after quench from HP experiments, and at lower T than previously reported. We describe the results of its characterization by FE-SEM, powder and single X-ray diffraction along with Raman spectroscopy, Nano-IR microscopy and Mössbauer spectroscopy techniques. Results are discussed in terms of effects on the density of the subducting slab and on redox interactions between slab and surrounding mantle components, with implications for the behavior of the slab at transition zone (TZ) and lower mantle (LM) depths and the origin of sub-lithospheric diamonds.

## Results

### Texture and composition

**Figure 1:**
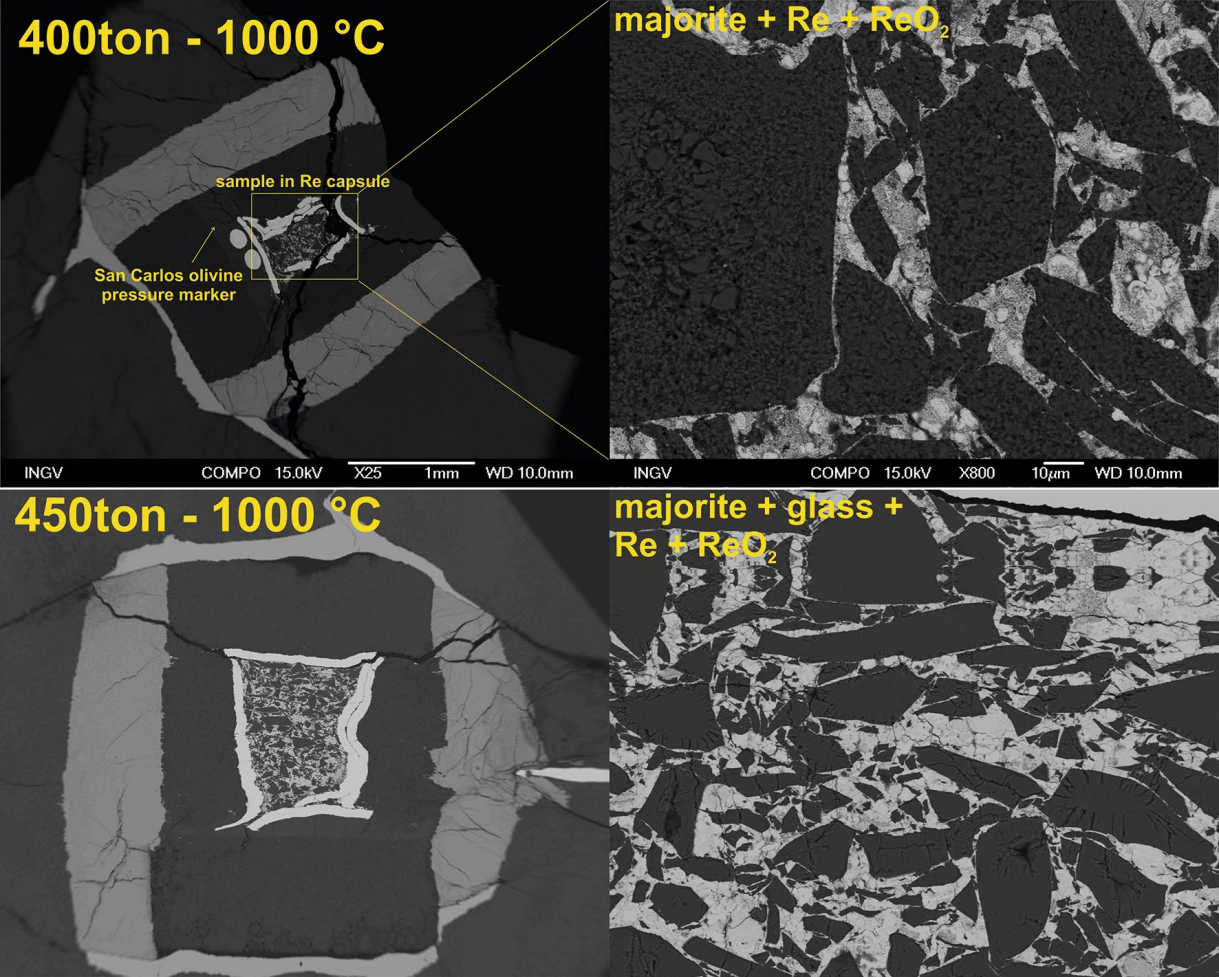
Back-scattered electron (BSE) images of the runs M81 (top—400ton–1000 °C) and M82 (bottom—450ton–1000 °C). Capsules and mineral phases crystallized in the charges are depicted in the images reported on the left and on the right, respectively.

A summary of the experimental conditions and recovered run products is reported in the Supplementary Information (Table S2) along with a sketch of the cell assembly used to reach the target pressure and temperature (Fig. S1). The chemical composition of the recovered samples is shown in Table 1. Results from these experiments are summarized in Fig. 1. These experiments were held for 30 min at 1000 °C and resulted in the presence of variably well-shaped grains of *Maj*, with sizes up to 100 µm also constituted by an aggregate of smaller crystals (run M81). No additional minerals were observed. The oxygen fugacity in these runs was buffered by the coexistence of both Re and ReO_2_ to allow comparison of the Fe^3+^/∑Fe ratio with that of previous studies (see “Discussion” section).

**Table 1:** Chemical composition in wt% of the recovered samples.

Run	N meas	Na_2_O	MgO	Al_2_O_3_	SiO_2_	CaO	Fe_2_O_3_	Total
M81	10	5.84 (72)	9.64 (30)	11.32 (37)	55.17 (27)	14.72 (69)	4.03 (38)	100.72 (1.02)
M82-rim	12	5.88 (45)	9.44 (42)	11.17 (29)	55.31 (81)	15.06 (62)	4.31 (41)	101.17 (1.09)
M82-core	7	5.87 (17)	9.44 (39)	10.97 (47)	54.86 (85)	15.13 (59)	4.27 (44)	100.54 (1.42)

**Figure 2:**
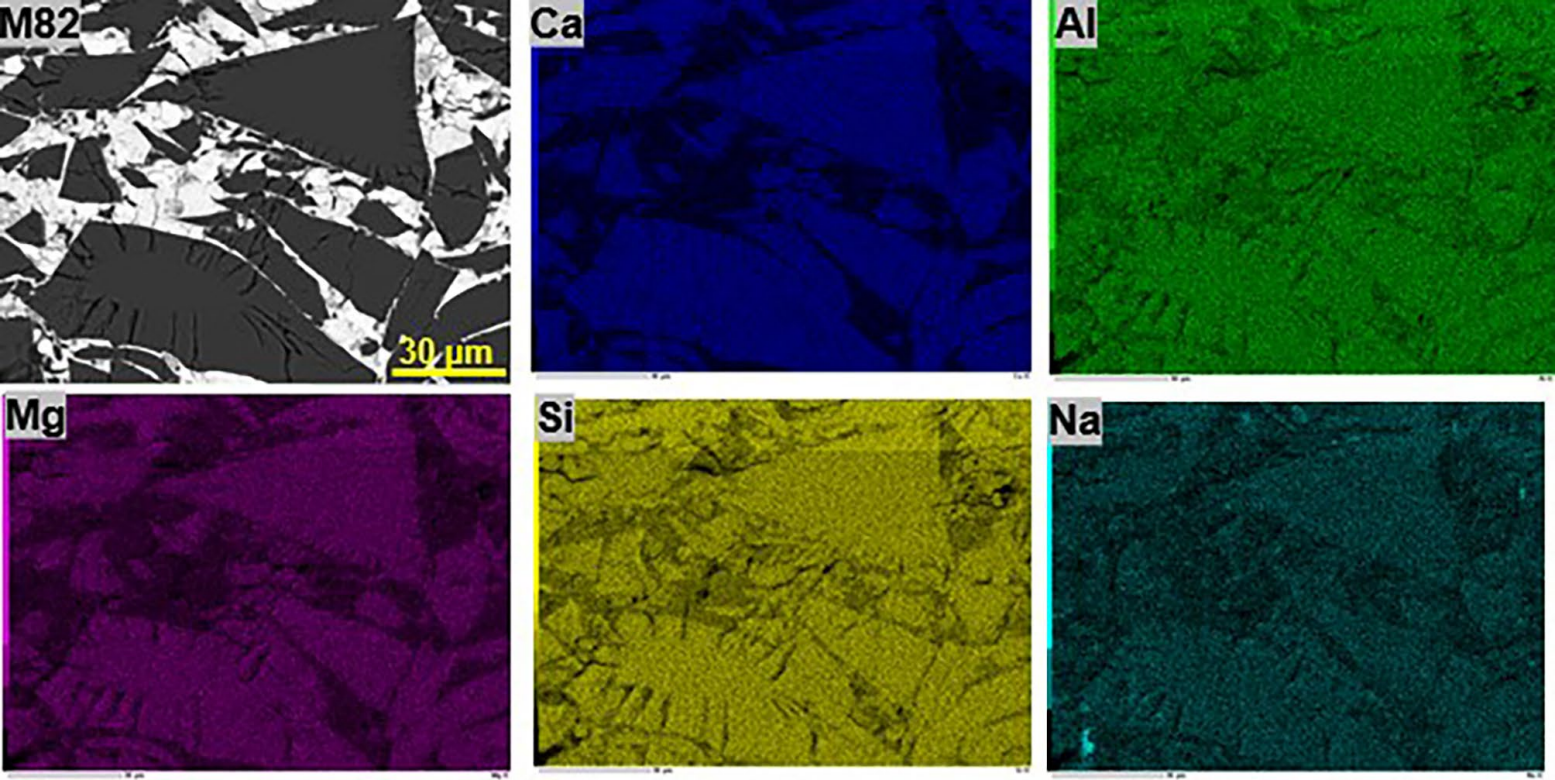
Chemical maps collected on sample M82 showing no differences of the chemical composition between the rim and core of the recovered phases.

In run M81, the mineral phases observed in the capsule with the SEM are *Maj* + Re^0^ + ReO_2_. In run M82, *Maj* rims the residual starting glass (Figs. 1 and 2). The recovered products were found to retain the same chemical composition of the initial *Cpx* glass with ~ 5.8 wt% Na_2_O content and 55 wt% SiO_2_ corresponding to ~ 20% Na-*Maj* (Na_2_MgSi_5_O_12_) and ~ 58% *Maj* (Mg_4_Si_4_O_12_) end-members. This can be seen in Fig. 2 where a map of chemical elements in the quenched products is shown.

### X-ray diffraction measurements and Mössbauer spectroscopy

The results from single-crystal XRD revealed that the phases in Fig. 1 consist of multiple crystallites. Only 4 single-crystal reflections (i.e., *d* = 1.54, 2.46, 2.58 and 2.89 Ả) belonging to the cubic *Grt* structure were unambiguously indexed giving *a* ~ 11.5 Å. On the other hand, the powder diffraction pattern collected on the same fragment showed the presence of several diffraction peaks (Fig. S2 of Supplementary Information), again all belonging to the cubic *Grt* structure. The least squares refinement of the diffraction peaks led to *a* = 11.4137(3) Å. Such a value is smaller than that observed for Na-bearing synthetic (Mg_2.802_Na_0.198_)(Al_1.790_Si_0.150_Mg_0.060_)Si_3_O_12_ *Grt* [*a* = 11.456(2) Å] studied by^38^, but it is larger than that measured for the Na-*Maj* (Na_2_MgSi_5_O_12_) end member^44^. Although synthetic Na_2_MgSi_5_O_12_ exhibits a tetragonal distortion, which is not observed in the present sample (no evidence of splitting of high-θ reflections), we can re-calculate an average cubic unit-cell parameter (*a* = 11.377 Å), which well reflects the decrease of the unit-cell volume with the increase of the amount of octahedral silicon. The ideal chemical formula of the crystal studied here, [CaNaMg][FeSiAl]Si_3_O_12_, verifies the pressure dependent exchange reaction Na^*X*^ + Si^*Y*^ = Mg^*X*^ + Al^*Y*^ ^19,40^ assumed in Na-bearing *Grt* on the basis of the obtained experimental data^38–44^. The average cubic unit-cell parameter of the quenched *Maj* is also smaller than the cell parameter determined for (Na_0.92_Mg_2.08_)(Mg_0.02_Al_1.06_Si_0.92_)Si_3_O_12_ (~ 49% En-5% pyrope-46% Jd), as synthesized within 1 h by ref. 47 at 22 GPa and 2000 °C pointing out, therefore, the effect of the chemical composition on the *Maj* crystal structure, while the P–T dependence on the lattice parameter remains to be investigated.

**Figure 3:**
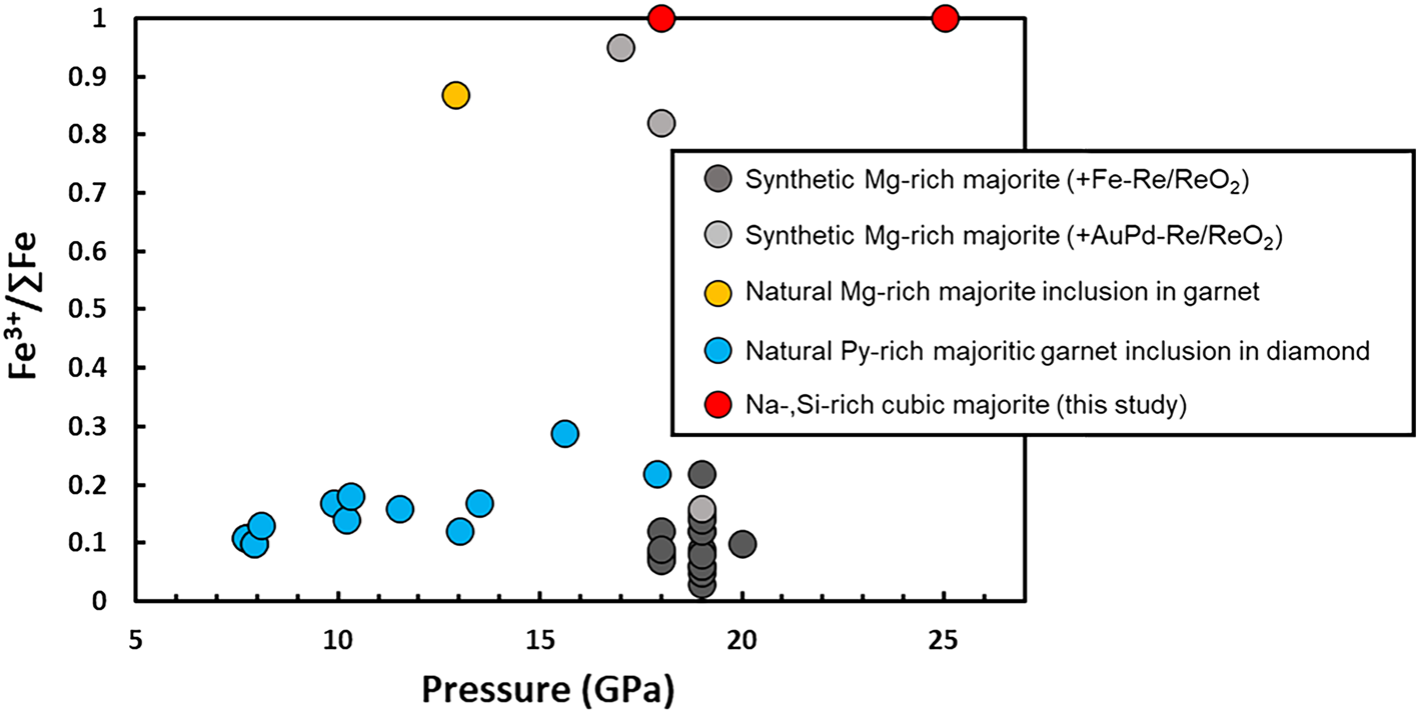
Variation of Fe^3+^/∑Fe with pressure for Na-rich *Maj* (this study) compared with literature data for majoritic *Grt* included in sub-lithospheric diamonds^29^, a natural eclogitic *Maj* included in *Grt*^48^, and synthetic peridotitic *Maj* under redox buffered conditions^49,50^.

Mössbauer spectra were collected on the quenched cubic *Maj* in runs M81 and M82 to investigate the Fe oxidation state. The quenched crystalline phases were found to contain entirely Fe^3+^, with a detection limit for Fe^2+^ estimated as 2% for M81 and 3% for M82. M81 and M82 spectra were fitted to one doublet for Fe^3+^ (Fig. S3). The hyperfine parameters for the Fe^3+^ doublet are consistent with those reported in the literature for both natural^29,48^ and synthetic^49,50^ tetragonal *Maj* (Fig. S4). A high Fe^3+^ content was previously observed in a natural *Maj* included in an eclogitic *Grt*^48^ and in synthetic hydrous *Maj*^50^ (Fig. 3). Conversely, some natural^29^ and synthetic^49^ majorites of peridotitic composition only show Fe^3+^ contents up to ~ 30%.

### Raman spectroscopy and nano-infrared microscopy

**Figure 4:**
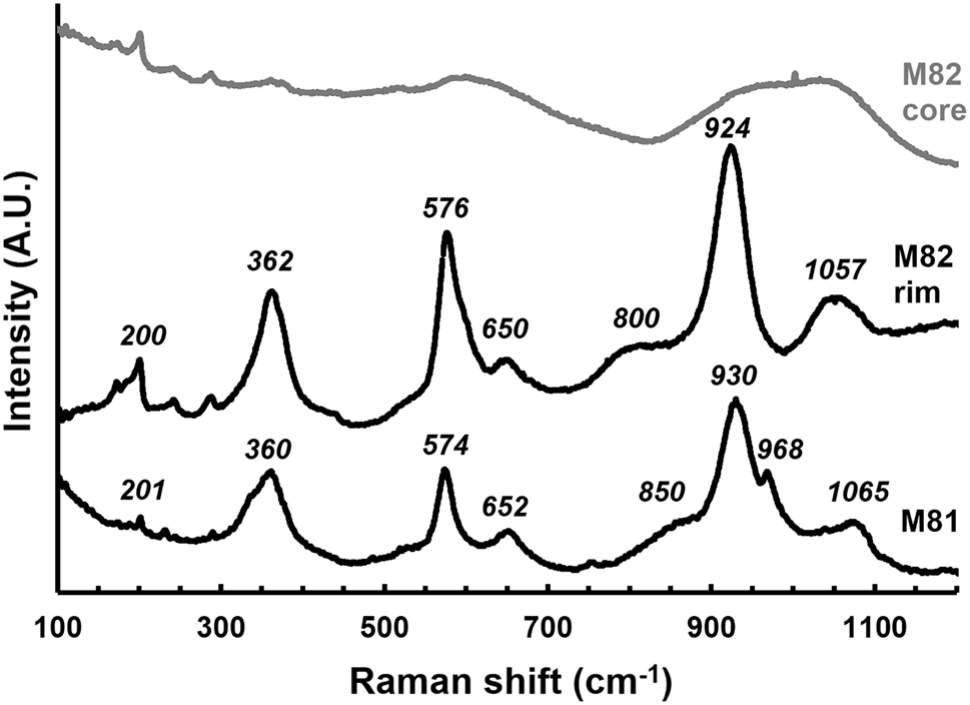
Raman spectra collected on samples M81, M82 core and M82 rim (in grey). The numbers reported above each peak indicate the Raman shift (in cm^−1^).

Strong evidence of the direct synthesis of cubic *Maj* from omphacitic glass is shown by the Raman spectra in Fig. 4 collected on M81, M82 core and M82 rim. The spectrum of *Maj* from run M81 appears similar to that from M82 rim, particularly regarding the positions and relative intensities of peaks at low to intermediate frequencies. By comparison with Raman frequencies reported by^51^, these Raman modes are assigned to dodecahedral translations (T_dod_, ~ 200 cm^−1^), A-type vibration due to rotation/libration (R, ~ 360 cm^−1^), symmetric (ν_2_, ~ 576 cm^−1^) and asymmetric bending motions (ν_4_, ~ 650 cm^−1^ and ~ 800–850 cm^−1^) of SiO_4_ tetrahedra. The high-frequency region is dominated by broad peaks due to symmetric (ν_1_, ~ 930 cm^−1^) and asymmetric (ν_1_, ~ 968 cm^−1^ in M81) SiO_4_ stretching modes, while the peaks at 1065 cm^−1^ in M81 and 1057 cm^−1^ in M82 from the rim are associated with Si–O stretching between linked SiO_4_ and SiO_6_ polyhedra, in agreement also with that found in the case of natural *Maj*^48^. Peak widths are very broad compared to naturally occurring *Maj*, which most likely reflects strong cation disorder^51,52^ typically observed in samples produced on a laboratory timescale. However, the extreme broadening observed in the M82 core spectrum (Fig. 4, gray line) is more of a testament to the amorphous, glassy nature of the starting material.

**Figure 5:**
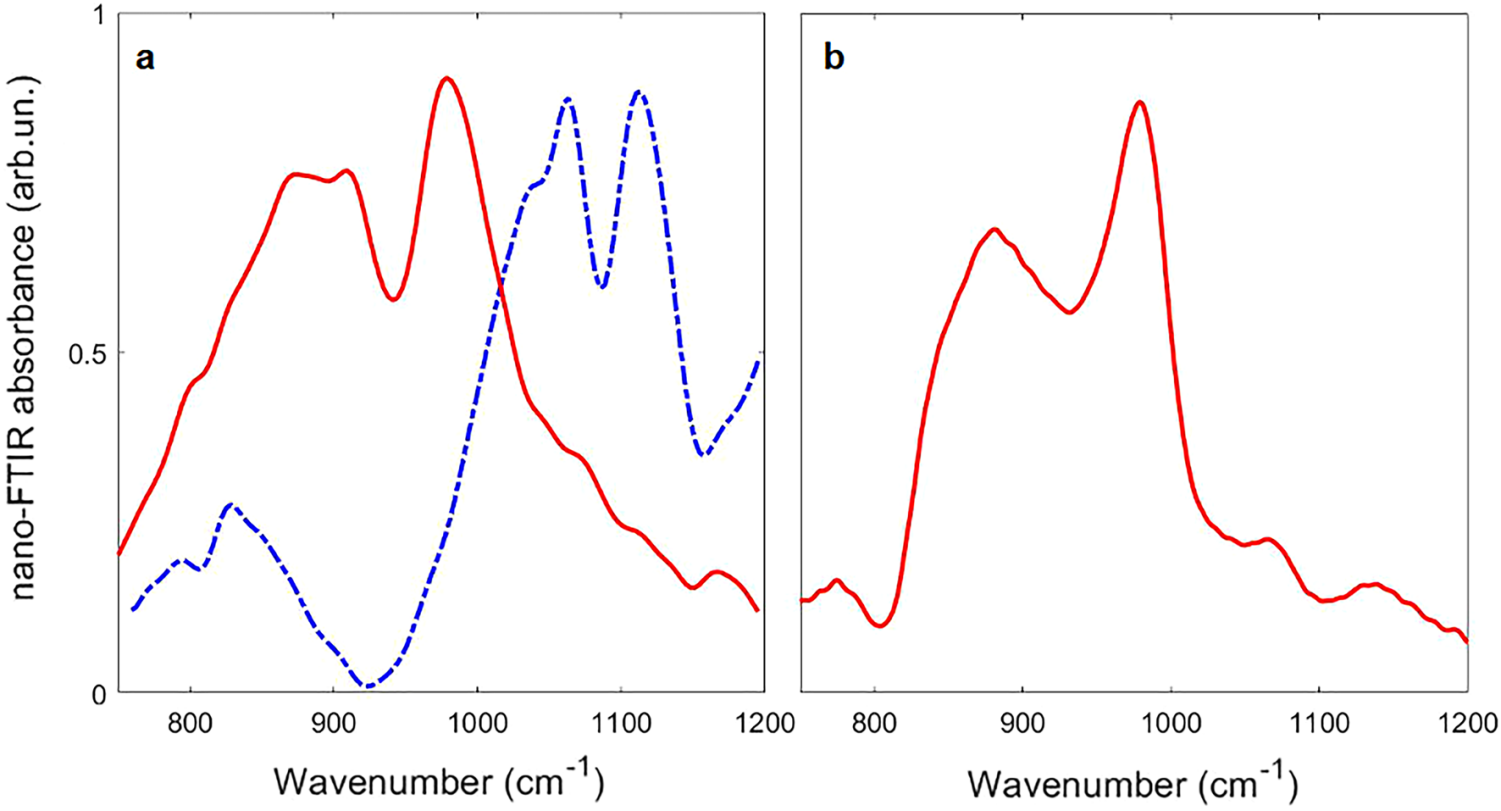
(**a**) Nano-Infrared spectra collected on two areas of samples M81 corresponding to the polycrystalline matrix (red continuous line) and the Re-ReO_2_ buffer (blue dotted line) for comparison. (**b**) Nano-Infrared spectrum collected on M81 polycrystalline matrix.

In view of the small grain size of the recovered quenched product and the absence of minor phases (e.g., exsolved minerals or amorphous unreacted portions), the homogeneous crystalline *Maj* product was further investigated by Nano-Infrared microscopy. Figure 5a,b shows spectra collected on M81 and M82 runs on an average of seven acquisitions on different spots. The absorption spectra of M81 and M82 in the spectral region between 750 and 1200 cm^−1^ are reported (with red continuous lines) respectively in Fig. 5a,b. An intense band between 850 and 1000 cm^−1^ is observed for both samples. In particular, three overlapping peaks are clearly detected for M81 at 870, 910 and 980 cm^−1^, while a peak at 880 cm^−1^ and two shoulders at 910 and 978 cm^−1^ are observed for M82. The discussed features are reproducible and match well with what found by author^51^ for *Maj*80. Remarkably, a previously unreported band is detected as a shoulder around 795 cm^−1^ in M81 spectrum and clearly observed as an isolated band for M82 at 774 cm^−1^. We suggest this to be characteristic of the Na-, Si- rich cubic *Maj* produced in the present study. These latter peaks must not be confused with that at ~ 785 cm^−1^ along with the peaks visible above 1000 cm^−1^ measured on the pockets filled by the Re-ReO_2_ buffer (blue dashed line in Fig. 5a).

## Discussion

### Some considerations on the crystal-chemistry of quenched cubic majorite

Our results show textural and chemical evidence of the formation of a cubic Na, Fe^3+^ and Si-rich *Maj* single phase crystallized from a reduced omphacitic glass resembling the chemical composition of eclogitic Na-rich *Cpx* within 15–30 min at estimated pressures of about 18 (M81) and 25 GPa (M82) at 1000 °C, thus extending our knowledge of the pressure-dependent exchange reaction Na^*X*^ + Si^*Y*^ = Mg^*X*^ + Al^*Y*^. In light of previous studies^45–47^, we hypothesize that the quenched synthetic Na, Si-rich phase is the product of isochemical phase transformation of metastable *Cpx*, which in nature occurs over the first ~ 200 km during subduction of cold slabs. The synthesized cubic Na, Si-rich *Maj* phase appears distinct from that reported in literature being Si^4+^  ~ 4 a.p.f.u., which is higher than 3.1–3.55 a.p.f.u. of *Maj* trapped in sub-lithospheric diamonds^29,53–57^, while the sum of 8-coordinated cations is 2.393 a.p.f.u. in M81 and 2.409 a.p.f.u. in M82, therefore lower than 2.7–3.4 a.p.f.u. of natural inclusions. Before the quenched phase can be equaled to the Si-rich *Grt* reported by authors^45–47^, several points must be considered. First of all, Gasparik and co-authors^45,46^ reported the occurrence of Si-rich *Maj* from a more ultramafic composition used as starting material that brought to the formation of additional minerals like Al-rich majorite coexisting with pyroxene and olivine (wadsleyite). In contrast, our runs resulted in the formation of Na,Si-rich *Maj* as single phase using an omphacitic glass as starting material. Secondly, Gasparik and co-authors^45,46^ reported much lower P (13.5, 14.5 and 15 GPa), higher T (1550–1650 °C) and longer runs (4 h) of equilibration for the Na,Si-rich *Maj*. In contrast, our experiments were conducted at higher P and much lower T and duration resulting in a well crystallized and chemically homogeneous *Maj* phase with cubic symmetry, taking into account that the aim of this study was neither to delineate the phase stability P–T field nor to constrain the kinetics of the *Cpx*—to—*Maj* transformation for which additional experiments are certainly needed. Finally, a cubic Na, Si-rich *Maj* was synthesized by ref.^47^ at 22 GPa and 2000 °C in 1 h, but with no Ca and Fe so that the composition cannot be considered representative of eclogitic *Cpx*. We conclude, therefore, that although a Na-, Si-rich cubic *Maj* has been shown to quench in this and a previous study within 15 min to 4 h, we cannot exclude the possibility that this mineral could have turned into either tetragonal Na-, Si-rich *Maj* or decomposed to a mineral assemblage in the case of much longer runs. Obviously, such an assessment would require time series experiments to demonstrate the long-time metastability of this polymorph. Here, we propose the origin of dominantly Na-rich *Maj* in a scenario where omphacite-rich lithologies persist during subduction and transform to a cubic Na, Si-rich *Maj* layer due to the absence or paucity of *Grt* as a reactant.

### Potential implications for the rheology of the subducted slab

To understand the potential effects of the omphacitic *Cpx* → cubic Na,Si-rich *Maj* transformation on the rheology of the subducted slab, we used our results to model the density, longitudinal (V_P_) and shear (V_S_) wave velocities of mid-ocean ridge basalt (MORB) at the pressures of the mantle transition zone (MTZ), using elastic parameters of major minerals available in the literature (Table 2). The elastic properties of the [CaNaMg][SiFeAl]Si_3_O_12_ cubic *Grt*, as well as MORB-like^58^ and pyrolite-like^59^ *Maj*, were estimated by a weighted summation of the individual elastic properties of Mg_3_Al_2_Si_3_O_12_ pyrope^60^, MgSiO_3_ majorite^61^, Ca_3_Al_2_Si_3_O_12_ grossular^58^, Ca_3_Fe_2_Si_3_O_12_ andradite^62^, Fe_3_Al_2_Si_3_O_12_ almandine^63^ and Na_2_MgSi_3_O_12_ majorite^64^. In the absence of direct measurements, the pressure and temperature dependences of the adiabatic bulk (*K*_*S*_) and shear (*G*) moduli of Na_2_MgSi_3_O_12_ *Maj* were derived from the elasticity dataset given by^41^. Comparison of our models for MORB and pyrolite *Maj* with experimental data allows to test the effect of non-ideal mixing on the elasticity of *Maj Grt* solid solutions. Our results show that this effect may be negligible at P higher than ~ 7 GPa (Fig. S5 in extended data) and therefore we assumed [CaNaMg][SiAl]Si_3_O_12_ cubic *Grt* is also behaving as an ideal solid solution at high pressure. Our model predicts that the longitudinal and shear velocities of [CaNaMg][SiAl]Si_3_O_12_ *Maj* are slightly higher than those of pyrolitic *Maj*^59^ while they are substantially lower than those of MORB *Maj*^58^ at the P conditions of the mantle transition zone (Fig. S5a,b).

**Table 2:** Elastic parameters of MORB-derived silicate minerals and end members.

Mineral	K_*S0*_ (GPa)	K_*S*_^*’*^	d*K*_*S*_/dT (GPa K^−1^)	*G*_*0*_ (GPa)	*G’*	d*G*/dT (GPa K^−1^)
CaMgSi_2_O_6_ diopside ^a^	116.4	4.9	− 0.012	73	1.6	− 0.011
Pyrope ^b^	170	4.5	− 0.017	93.2	1.51	− 0.011
Grossular ^c^	171	4.4	− 0.013	108	1.3	− 0.011
Andradite ^d^	154	4.7	− 0.013*	90	1.2	− 0.011*
Almandine ^e^	174	4.6	− 0.027	95	1.1	− 0.013
MgSiO_3_ *Maj* ^f^	164	4.1	− 0.022	86	1.1	− 0.009
Na-*Maj* ^g^	173	4.4*	− 0.013**	115	1.5	− 0.010**
MORB *Maj* ^h^	155.8	4.5	− 0.013**	89.7	1.5	− 0.010**
Pyrolite *Maj* ^i^	164.4	4.2	− 0.013	94.9	1.1	− 0.010
SiO_2_ + 2wt% Al_2_O_3_ Stishovite ^j^	286	5.2	− 0.045	199	2.5	− 0.017

a. *Ref.* ^90^, b. *Ref*. ^60^, c. *Ref*. ^91^, d. *Ref*. ^62^, e. *Ref*. ^63^, f. derived from *Ref*. ^61^, g. *Ref*. ^42,64^, h. *Ref*. ^58^, i. *Ref*. ^59^, j. *Ref*. ^92^. Value fixed to those of *grossular *Grt* and ** pyrolitic *Maj*, respectively. (+) *K*_*S*_ and *G* were taken from ref. ^64^, which has the closest composition to our cubic majorite. *Ks*’ was derived from *Kt’* of ^42^ and *G’* from assuming a constant Poisson ratio. Thermal parameters were assumed = pyrolitic *Maj.*

**Figure 6:**
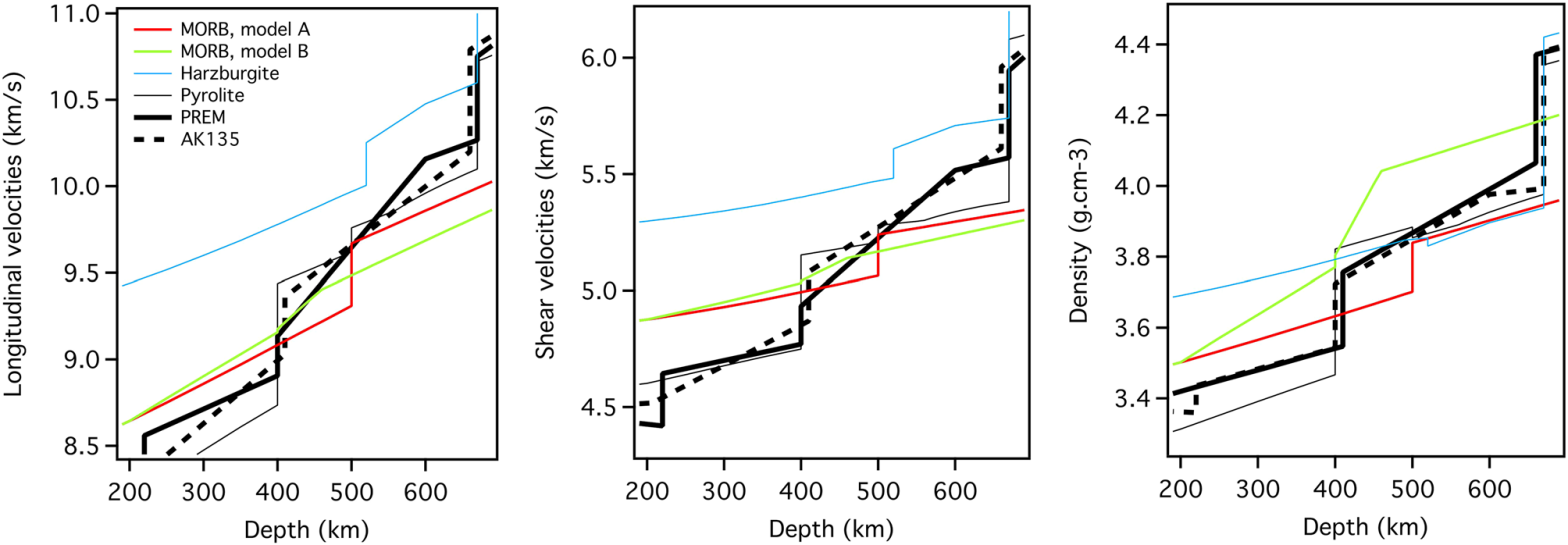
(**a**) Longitudinal, (**b**) Shear velocities and (**c**) density of MORB aggregates along a cold slab geotherm [ref.^66^] as a function of depth. Red line represents the disequilibrium MORB where the transition of *Cpx* to [CaNaMg][SiAl]Si_3_O_12_ majoritic *Grt* was fixed to 520 km depth. Green line represents equilibrium MORB where *Cpx* dissolved gradually in *Grt*, increasing in turn proportions of MORB majoritic *Grt*. Thin black line represents velocities and density of pyrolite for comparison, as well as seismological 1D models, PREM (thin black line) and AK135 (broken black line).

The elasticity of MORB aggregates was derived through a Voigt-Reuss-Hill (VRH) average of the individual mineral elastic properties, assuming relative proportions of each phase from phase equilibrium data proposed by^65^. In this calculation, we used a cold slab geotherm with an adiabatic temperature gradient as proposed by Thompson^66^, and which has a root temperature T = 280 K at 0 km depth (e.g., T = 1140 K at ~ 500 km depth). The calculated longitudinal velocities, shear velocities and density of MORB aggregates along a cold slab geotherm as a function of depth are shown in Fig. 6a–c. In the UM, we hypothesize a MORB-derived eclogitic slab that consists of 75 vol% *Cpx*, 20 vol% cubic *Grt* (e.g., almandine-pyrope-grossular solid solution) and ~ 5 vol% stishovite. Although the modal composition is strictly controlled by P, T and composition^14,32^, a representative seismic model can be drawn with the UM *Grt* being calculated following weighted average methods assuming a solid solution (in mol%) of 43% pyrope, 27% grossular and 30% almandine as suggested by the phase relation of MORB at 5 GPa and 1200 °C^67^. When equilibrated, the MORB assemblage transforms to majoritic *Grt* (95%) and stishovite (5%) upon the gradual dissolution of pyroxenes into the *Grt* at P between 7 and 15 GPa (Model B of Fig. 6). In contrast, at conditions where metastable minerals might be present such as those in the subducted cold slab, our results raise the question whether the assemblage of *Cpx*, *Grt* and stishovite remains stable up to ~ 18 GPa where omphacitic *Cpx* is here shown to transform directly to cubic [CaNaMg][SiAl]Si_3_O_12_ majoritic *Grt*, thus forming an assemblage of 75% majoritic *Grt*, 20% cubic *Grt* and 5% stishovite in the MTZ (Model A, Fig. 6). If this is the case, the transition of omphacitic *Cpx* to [CaNaMg][SiAl]Si_3_O_12_ cubic majoritic *Grt* at ~ 520 km depth would be accompanied by a ~ 0.2 g cm^−3^ density increase while V_P_ and V_S_ would increase by 0.5 and 0.2 km s^−1^, respectively (Fig. 6). These results inferred from our experiments show that unequilibrated MORB mineral assemblages would have density similar to that of the surrounding mantle and harzburgite (blue line in Fig. 6), hence, forcing the slab to float between 500 and 660 km depth. These results are strengthened by harzburgite, which constitutes the main body of the subducted slab, and whose density is even further below those of pyrolite while yielding higher velocities^59,68,69^. The presence of harzburgite and unequilibrated MORB mineral assemblages (with the hypothesized proportions) might have favored regimes of slab floating at middle-to-lowermost MTZ (see Fig. 6c) for millions of years such as those observed by seismic tomography beneath Europe or North America^70^. Obviously, an experimental study aimed to investigate the kinetics of the cubic-to-tetragonal majorite transformation is needed to strengthen such a conclusion. In contrast, equilibrated MORB mineral assemblage (green line in Fig. 6c) would cause the slab to sink into the LM as the density is far higher than those of pyrolite and harzburgite in the MTZ^71^. Importantly, after transformation of omphacitic *Cpx* to *Grt*, only V_P_ contrast appears significant while V_S_ are almost identical. Finally, as dense majorite MORB forms upon equilibration of the slab (e.g., model A transforms to model B), this sinks down into the LM carrying, therefore, incompatible and refractory elements such as Na and Al, respectively.

### Majorite as diamond growth-medium

The calculated proportions of Na-majorite component, Na_2_MgSi_5_O_12_ [(Na)/2], and majorite component, Mg_3_MgSiSi_3_O_12_ [(Si-2Na-*Maj*)-3] can be used as a geobarometer^19^. From our experiments, we calculated proportions of 0.20 and 0.57, respectively, for the two components giving a pressure estimate of ~ 20 GPa. The Mg# [(Mg/Mg + Fe^2+^)] of 0.81–0.83 and Ca# [Ca/(Ca + Mg + Fe^2+^)] of 0.47–0.48 (Fe^2+^ recalculated from the measured Fe^3+^) are both intermediate with respect to Mg# and Ca# measured in natural majoritic inclusions with peridotitic and eclogitic affinity (see Fig. 7a,b in Ref.^19^). Conversely, the unique chemical composition of our synthesized *Maj* is very similar to that reported for two mineral inclusions (BZ237A and BZ259B) found in a diamond from Juina (Brazil)^72^ and referred to a III-type mineral association representative of the TZ and LM. Indeed, the chemical composition of these inclusions match well with our quenched cubic *Maj* much more than that of another diamond inclusion from the kimberlite pipe in Liaoning Province (China)^73^. In this latter case, the mineral was proposed to contain 16 mol% Na-*Maj* and 84 mol% Mg-*Maj*, corresponding to 2.3 wt% Na_2_O and 33 wt% MgO. Experiments at HP-T suggested the possible origin of this Na-rich *Maj* as unmixing of Al-rich *Maj* and as reaction product of MgSiO_3_ bridgmanite with an alkali-rich carbonatitic melt at depths the TZ and LM^74,75^. However, the temperature of these experiments is unfeasibly high (1900–2000 °C) within the context of any plausible subduction scenario. We emphasize that the new synthesized cubic *Maj* contains about 5.8 wt% Na_2_O, more than 9 wt% MgO, along with CaO (~ 14–15 wt%), Al_2_O_3_ (~ 11 wt%) and Fe_2_O_3_ (~ 3.7 wt%), and it was quenched at P representative of the TZ and LM depths. The lack of evidence for cubic Na-, Si- rich majorite inclusions in diamonds with chemical compositions similar to that reported in this study can be explained as a consequence of the long equilibration times required to precipitate diamonds compared to the those of synthesis reported here. Noteworthy, the high Fe^3+^ content of cubic *Maj* implies a major role in redox reactions at the expense of oxidized C-bearing melts to precipitate diamonds via the chemical equilibrium,

1 $${\text{6Fe}}\left( {^{{{2} + }} } \right){\text{O}}_{Maj} + {1}.{\text{5CO}}_{{{\text{2melt}}}} = {\text{3Fe}}\left( {^{{{3} + }} } \right)_{{2}} {\text{O}}_{{{3}Maj}} + {1}.{\text{5C}}_{{{\text{diamond}}}}$$

### On the kinetics of cubic-to-tetragonal majorite

It is known that cubic *Maj* with composition Wo_1_En_78_Fs_21_ was first found within minerals in shock impact in meteorites^76^. An accurate literature search reveals that majoritic *Grt* from shocked chondrites^76–81^ are characterized by Si ranging from 3.55 to 3.99 a.p.f.u., with the highest contribution to the majoritic end member given by the low-Al *Grt* that transformed directly from enstatite, whereas the more Al-rich compositions refers to *Grt* that crystallized from a shock-induced melt. Regardless, all of these majorite garnets are characterized by a cubic symmetry explained as a consequence of either the rapid quench on the atomic ordering (requiring longer time than a metastable phase transition) or their relatively high Fe/(Fe + Mg) ratios of 0.20–0.27 responsible for the stabilization of the cubic structure^82^. The incorporation of elements such as Fe^3+^ and Na, as well as the T effect were indicated as likely to further stabilize the cubic structure of *Maj* in the TZ^82–84^. Our current results would confirm these expectations.

## Materials and methods

### Synthesis

The starting material used in this study is a synthetic omphacitic glass with composition as reported for natural Bavarian (Weissenstein) eclogite^85^ (COMP2, ref.^27^). The synthetic omphacitic glass was prepared by melting a mixture of oxides and carbonates at 1650 °C in an iron-saturated platinum crucible followed by rapid quenching in water and ice. The glass was analyzed using the electron microprobe (details in ref.^27^) and then ground and reduced in a gas mixing furnace at 800 °C using a H_2_-CO_2_ gas mixture at a *f*o_2_ of ~ 2 log units above the iron-wüstite buffer. The glass was, then, powdered and mixed with 25% of a Re and ReO_2_ (1:1 mol ratio) mixture to act as an oxygen buffer. Two experiments were performed at pressures of ~ 18, and ~ 25 GPa at 1000 °C using the 840-ton Walker-type press available at the High-Pressure High Temperature laboratory of the National Institute of Geophysics and Volcanology (INGV, Italy). Tungsten carbide anvils (F grade) with 3 mm truncation edge lengths (TEL) were used with cobalt-doped MgO octahedra as pressure media. Rhenium capsules were employed in our experiments to prevent loss of Fe from the starting material during the experiments. After loading with the starting powder, the capsule was placed in the central portion of a cylindrical straight LaCrO_3_ furnace separated by MgO sleeve. MgO disks as spacers were placed at the bottom and top of the capsule to serve as pressure medium. The temperature during the experiments was monitored with a W-5%Re/W-26%Re (C-type) thermocouple inserted through a hole drilled in the pressure medium and the heater above which the capsule was located. The gaps between the thermocouple and the capsule were filled with olivine (San Carlos) powder used as pressure marker based on the olivine-ringwoodite and ringwoodite-periclase + bridgmanite phase transitions^86^. The sample was compressed to the target pressure at a rate of ~ 0.5 GPa/h, then heated and kept at a constant temperature within ± 10 °C for a period of 30 min. The run was quenched by turning off the power to the furnace and then, decompressed to ambient pressure within 15 h.

### Field emission scanning electron microscopy

Textural observations and quantitative chemical compositions of the run products were performed using a JEOL JSM-6500F field emission scanning electron microscope at the Microanalysis Lab of National Institute of Geophysics and Volcanology (INGV, Rome). The FE-SEM apparatus is equipped with back-scattered electron detector and energy dispersion system (JEOL 133 eV resolution), and the operative acquiring conditions established for accurate analyses are an accelerating voltage of 15 kV and a probe current of 0.8 nA. Samples were C-coated before being analyzed.

### X-ray diffraction

X-ray diffraction investigations were performed at the CRIST, Centro di Studi per la Cristallografia Strutturale, Università di Firenze, Italy. A small fragment (size about 17 × 22 × 34 μm^3^) was extracted from the polished section of one of the recovered samples (M82) under a reflected light microscope and mounted on a 5 µm diameter carbon fiber, which was, in turn, attached to a glass rod. The single-crystal X-ray study was done with an Oxford Diffraction Xcalibur3 CCD single-crystal diffractometer using MoKα radiation (λ = 0.71073 Å), working conditions 60 kV × 50 nA and with 300 s exposure time per frame; the detector-to-sample distance was 6 cm. Then, to get a powder diffraction pattern, the same grain was studied with an Oxford Diffraction Xcalibur PX Ultra diffractometer equipped with a 165-mm diagonal Onyx CCD detector at 2.5:1 demagnification operating with CuKα radiation (λ = 1.5406 Å). The working conditions were 50 kV × 50 nA with 7 h of exposure; the detector-to-sample distance was 7 cm. Data were processed using the CrysAlis software package version 1.171.36.28 running on the Xcalibur PX control PC.

### Mössbauer spectroscopy

Fe^3+^/ΣFe ratios were estimated using Mössbauer spectroscopy. Octahedra from high-pressure runs were mounted in epoxy resin and cut into slices to expose the sample on both sides. Slices were double polished to a thickness of 600 μm, which is close to the optimum thickness based on sample composition^87^ and corresponds to a dimensionless effective thickness of 2 (5 mg Fe/cm^2^). A region of recovered products containing majorite with dimensions of 800 μm × 600 μm was exposed on each side of sample slices, and the outside area was covered with 25 μm thick Ta foil, which absorbs 99% of 14.4 keV gamma rays. Mössbauer spectra were recorded at room temperature in transmission mode on a constant acceleration spectrometer using a nominal 370 MBq ^57^Co point source at Bayerisches Geoinstitut, Bayreuth. The collecting time for each spectrum was 2 weeks. The velocity scale, set at ~ 5 mm/s, was calibrated relative to an α-Fe foil reference standard. Once folded, spectra were fitted to Lorentzian line-shapes using the fitting program MossA^88^.

### Raman spectroscopy and nano-infrared microscopy

The crystallinity of the recovered products was checked by Raman spectroscopy using a Horiba Jobin Yvon LABRAM HR800 spectrometer at the Experimental Volcanology and Petrology Laboratory (EVP Lab) (University of Roma Tre, Rome). The spectrometer is equipped with two gratings (1800 and 600 grooves/mm), a CCD detector (operating at − 70 °C), an Olympus optical microscope (objectives 10X, 20X, 50X and 100X) and a solid-state Nd-YAG laser as source (wavelength 532 nm, power 60 Mw).

Nano-Infrared Microscopy measurements were carried out at the SISSI beamline of Elettra/CNR-IOM at Elettra Sincrotrone (Basovizza, Trieste, Italy) by using a NEASPEC s-SNOM instrument. Measurements were performed in tapping mode at a tapping frequency of 260 kHz. Tapping amplitude was set to 80 nm (with approach at 80% of free amplitude). Spectra were acquired at a resolution of 6 cm^−1^. A N_2_-cooled MCT (Mercury–Cadmium–Tellurium) detector was used to detect infrared signal. The absorption was calculated directly from Neaspec acquisition software following the procedure described in ref.^89^.

### Supplementary Information

Supplementary Information.

## Data Availability

The datasets used and/or analysed during the current study are available from the corresponding author on reasonable request.
