# Supplementary material for: Cubic Fe-bearing majorite synthesized at 18–25 GPa and 1000 °C: implications for element transport, subducted slab rheology and diamond formation
Source: Sci Rep. 2023 Sep 22;13:15855. doi: 10.1038/s41598-023-43037-6 (PMC10516933; doi:10.1038/s41598-023-43037-6)
Supplement: Supplementary file 1 — Supplementary Information. [file 41598_2023_43037_MOESM1_ESM.docx]

Supplementary information for

**Cubic Fe-bearing majorite synthesized at 18-25 GPa and 1000 °C: implications for element transport, subducted slab rheology and diamond formation**

Vincenzo Stagno^1,9*^, Luca Bindi^2^, Barbara Bonechi^3^, Steeve Greaux^4,5^, Sonja Aulbach^6^, Tetsuo Irifune^4,5^, Stefano Lupi^7,10^, Giulia Marras^1^, Catherine A. McCammon^8^, Manuela Nazzari^9^, Federica Piccirilli F.^10^, Brent Poe^11^, Claudia Romano^12^, Piergiorgio Scarlato^9^

^1^Dipartimento di Scienze della Terra, Sapienza Università di Roma, Roma, Italy.

^2^Dipartimento di Scienze della Terra, Università di Firenze, Firenze, Italy.

^3^University of Manchester, Manchester, United Kingdom.

^4^Geodynamic Research Center, Ehime University, Matsuyama, Japan.

^5^Earth-Life Science Institute, Tokyo Institute of Technology, Tokyo, Japan.

^6^Goethe University, Institute for Geosciences; Frankfurt Isotope & Element Research Center (FIERCE), Altenhöferallee 1, 60438 Frankfurt, Germany.

^7^Department of Physics, Sapienza University of Rome, 00185 Rome, Italy.

^8^Bayerisches Geoinstitut, Bayreuth Universität, Bayreuth, Germany.

^9^Istituto Nazionale di Geofisica e Vulcanologia, Roma, Italy.

^10^CNR-IOM, Area Science Park, I-34012 Trieste, Italy.

^11^Dipartimento di Scienze, Università di Chieti, Chieti, Italy.

^12^Dipartimento di Scienze, Università di Roma Tre, Roma, Italy.

*Corresponding author: Vincenzo Stagno ([vincenzo.stagno@uniroma1.it](mailto:vincenzo.stagno@uniroma1.it)).


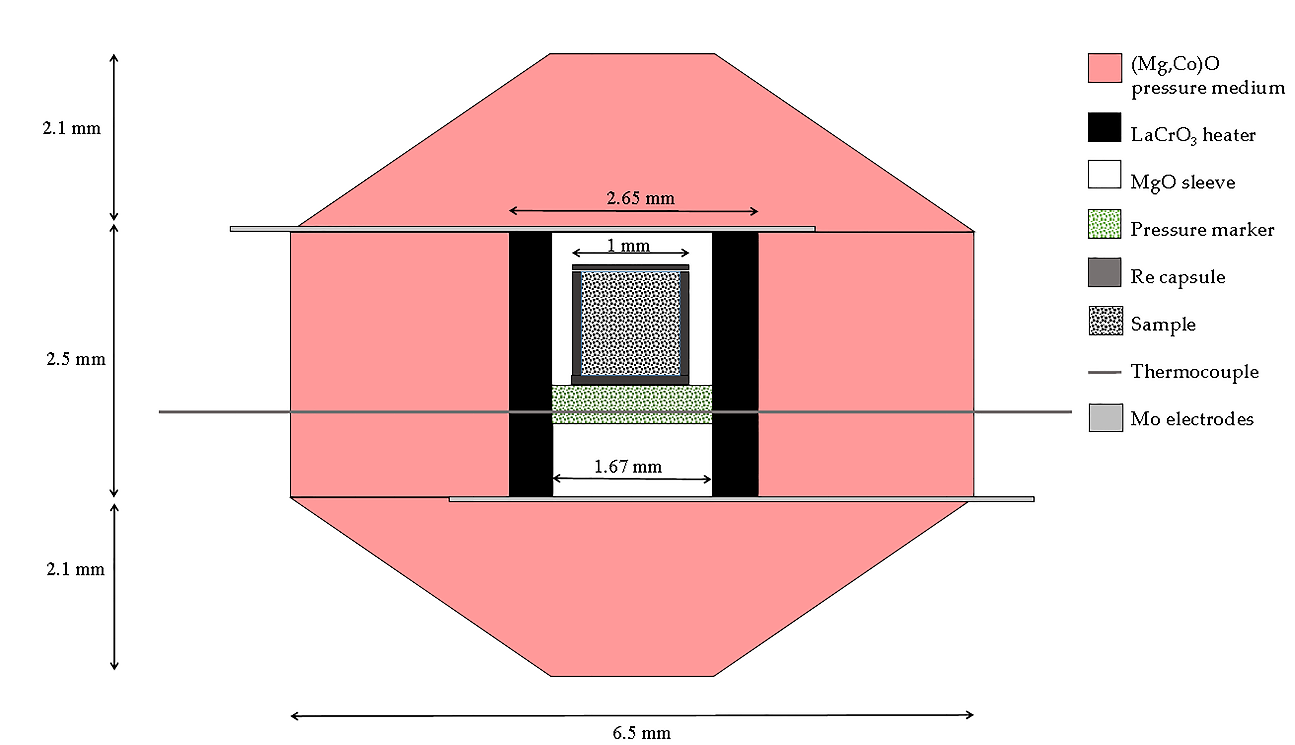


Figure S1. Schematic cross section of the cell assembly used in this study.


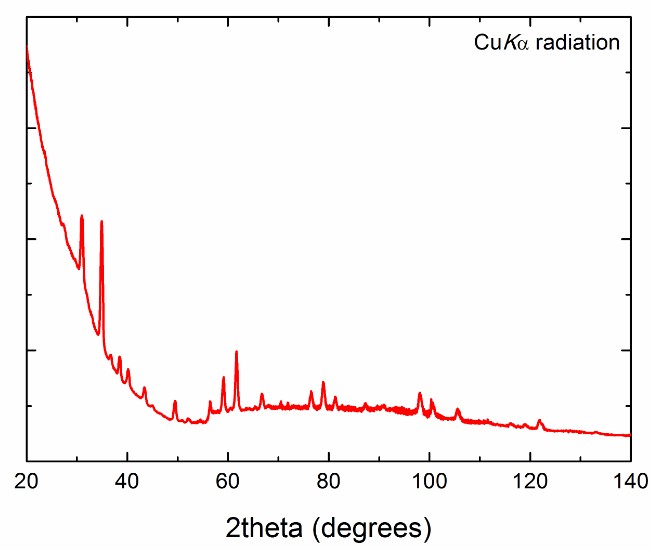


Figure S2. X-ray powder diffraction pattern collected from the majorite fragment (run M82). All peaks can be assigned to cubic majorite.


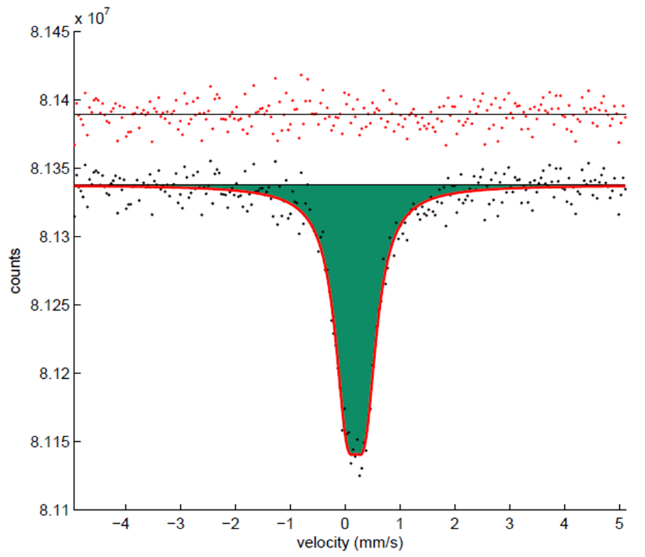

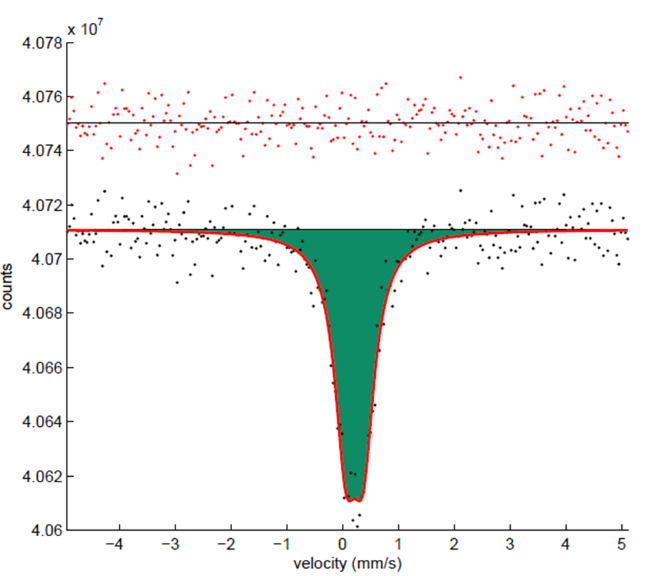


***M82***

***M81***

Figure S3. Fitted Mössbauer spectra showing the presence of Fe all as Fe^3+^. Spectra were fitted to one doublet.


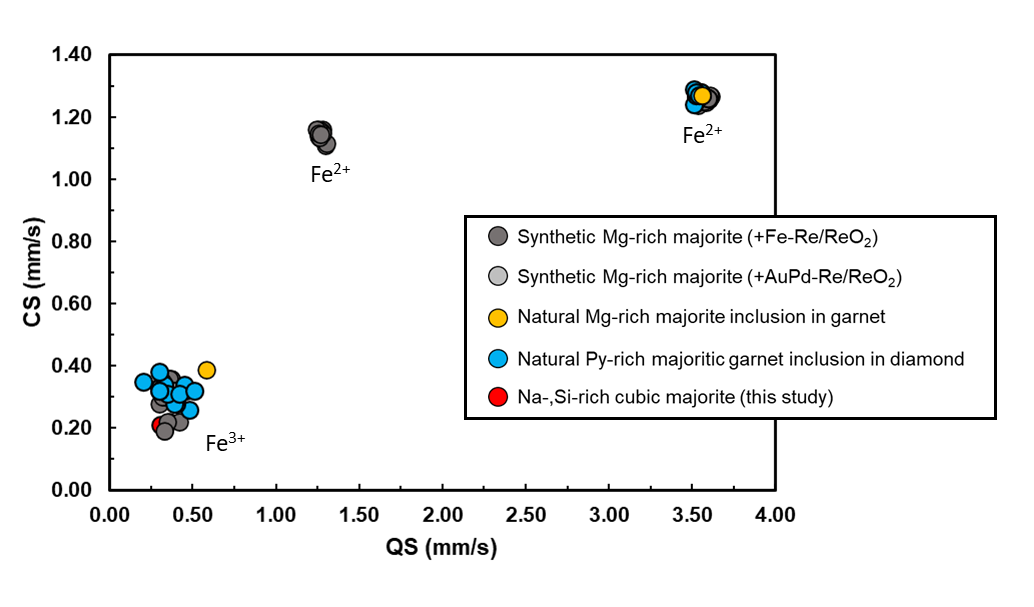


Figure S4. Hyperfine parameters (center shift vs quadruple splitting) from our study plotted along with literature data for both natural^28,48^ and synthetic^49,50^ majorites.

b.

a.

Figure S5. a) Longitudinal and b) Shear velocities of MORB *Grt* solid solutions as a function of pressure, at 300 K. Plain lines represent our calculated velocities for [CaNaMg][SiAl]Si_3_O_12_ *Maj* (red), pyrolitic (blue) and MORB (thick black) *Maj Grt*, and an UM MORB *Grt* (thin black) with an assumed composition (in wt.%) of 43% pyrope, 27% grossular and 30% almandine (e.g. ref. 60, Table 3, 5 GPa, 1200 °C). The open square and circle symbols represent the corresponding experimental data for the pyrolitic and MORB majorite *Grt*; Our calculated V_P_ and V_S_ are fairly consistent with those of experimental studies at P > 10 GPa, which corresponds to the depths of the mantle TZ.

**Table S1. Chemical composition of the glass used as starting material.**

| **This study**  **WM6.80** | | **Bobrov et al.^39^** |
| --- | --- | --- |
|  | **Wt%**  **Cpx [15]** | **Wt%**  **Di_40_Hd_10_Jd_50_** |
| SiO_2_ | 54.60(42) | 56.55 |
| MgO | 9.43(9) | 7.59 |
| Al_2_O_3_ | 10.52(8) | 12 |
| CaO | 15.30(17) | 13.20 |
| FeO_tot_ | 3.36(4) | 3.38 |
| Na_2_O | 5.63(31) | 7.29 |
| TiO_2_ | 0.17(2) | - |
| Total | 99.01(44) | 100 |

*Notes:* In brackets is the number of points analysed by electron microprobe. WM6.80Cpx is synthetic glass with composition similar to natural *Cpx* reported by Stosch and Lugmair^85^. Errors in parentheses are 1σ.

**Table S2**. Experimental run conditions.

| **Run** | **P (ton)** | **T(°C)** | **Duration (min)** | **Pressure marker** | **Phases** |
| --- | --- | --- | --- | --- | --- |
|  |  |  |  |  |  |
| M81 | 400 | 1000 | 15 | rwd | Cubic majorite |
| M82 | 450 | 1000 | 30 | rwd + bgm (+ per) | Cubic majorite |
|  |  |  |  |  |  |

rwd = ringwoodite; maj = majorite; per = periclase; bgm = bridgmanite
